# Reconstructing the value puzzle in health technology assessment: a pragmatic review to determine which modelling methods can account for additional value elements

**DOI:** 10.3389/fphar.2023.1197259

**Published:** 2023-07-13

**Authors:** Jeffrey M. Muir, Amruta Radhakrishnan, Andreas Freitag, Ipek Ozer Stillman, Grammati Sarri

**Affiliations:** ^1^ Cytel Inc., Toronto, ON, Canada; ^2^ Cytel Inc., London, United Kingdom; ^3^ Takeda Pharmaceuticals, Lexington, MA, United States

**Keywords:** cost-effectiveness analysis, value elements, health technology assessment, health policy, societal value, health equity

## Abstract

Health technology assessment (HTA) has traditionally relied on cost-effectiveness analysis (CEA) as a cornerstone of evaluation of new therapies, assessing the clinical validity and utility, the efficacy, and the cost-effectiveness of new interventions. The current format of cost-effectiveness analysis, however, does not allow for inclusion of more holistic aspects of health and, therefore, value elements for new technologies such as the impact on patients and society beyond its pure clinical and economic value. This study aimed to review the recent modelling attempts to expand the traditional cost-effectiveness analysis approach by incorporating additional elements of value in health technology assessment. A pragmatic literature review was conducted for articles published between 2012 and 2022 reporting cost-effectiveness analysis including value aspects beyond the clinical and cost-effectiveness estimates; searches identified 13 articles that were eligible for inclusion. These expanded modelling approaches mainly focused on integrating the impact of societal values and health equity in cost-effectiveness analysis, both of which were championed as important aspects of health technology assessment that should be incorporated into future technology assessments. The reviewed cost-effectiveness analysis methods included modification of the current cost-effectiveness analysis methodology (distributional cost-effectiveness analysis, augmented cost-effectiveness analysis, extended cost-effectiveness analysis) or the use of multi-criteria decision analysis. Of these approaches, augmented cost-effectiveness analysis appears to have the most potential by expanding traditional aspects of value, as it uses techniques already familiar to health technology assessment agencies but also allows space for incorporation of qualitative aspects of a product’s value. This review showcases that methods to unravel additional value elements for technology assessment exist, therefore, patient access to promising technologies can be improved by moving the discussion from “if” to “how” additional value elements can inform decision-making.

## Introduction

Value in health technology assessment (HTA), which is the foundation upon which decision-making regarding new drugs and health technologies is made in several healthcare systems, has been primarily based on balancing the clinical benefits to patients and/or economic costs involved by introducing the new technology to the healthcare system. Expansion of the concept of value in HTAs has been the subject of recent research and debate mainly driven by patients, carers and clinicians who recognize that the value of a new technology is multidimensional (Caro et al., 2019; Reed et al., 2019). This multidimensional nature is reflected in the latest definition of HTA provided by the Professional Society for Health Economics and Outcomes Research (ISPOR) Task Force which, in part, notes that dimension of value for a health technology may be assessed “by examining the intended and unintended consequences of using a health technology” and that this evaluation should encompass a comprehensive array of factors, including ethical, social, and cultural issues (O'Rourke et al., 2020a; O'Rourke et al., 2020b).

To this end, several organizations and research groups have developed value-based frameworks as an attempt to address the limitations of current HTA decision tools (Zhang et al., 2022). The ISPOR Strategic Task Force is leading an effort to reshape the future of HTA by examining the definition of a technology’s value and encouraging the integration of additional elements of value not currently included in the technology submissions. The findings regarding new concepts of value have been summarized in the ISPOR Task Force’s “Value Flower” (Lakdawalla et al., 2018). Some of the proposed elements beyond the traditional clinical and cost-effectiveness analyses include the value of: the reduction in uncertainty surrounding a disease, the fear of contagion, the value of insurance, the severity of disease, the value of hope, real option value, health equity, and scientific spillovers (Lakdawalla et al., 2018). Indeed, the Second Panel on Cost-effectiveness (Sanders et al., 2016) has recommended the incorporation of reference cases in each cost-effectiveness analysis (CEA) and an “impact inventory,” i.e., a cataloguing of consequences of analysis decisions both inside and outside of the healthcare sector. Previous research has also shown that even though these expanded value-based frameworks (generic or disease specific) provide the possibility of incorporating additional benefits that technologies may bring to patients and society and contextual factors to be considered through deliberative processes, there are practical limitations for their implementation in routine HTA decision-making (Willke et al., 2018; Breslau et al., 2023). One of the main barriers for the wider implementation of these value-based frameworks in decision-making, especially when CEA is the pillar of HTA, is the lack of consensus on how reliably and consistently these elements can be applied across different disease indications and technologies (Willke et al., 2018; Reed et al., 2019). Additionally, the lack of consensus regarding methods to address these concerns, the concerns of double counting of outcomes or interdependent variables raised by this lack of consensus and the historically narrow remit of HTA agencies (i.e., costs and benefits are assessed from a healthcare systems or payer perspective) represent significant barriers to widespread adoption (Fornaro et al. 2021; Hendriks and Pearson, 2021; Garrison et al., 2020). As a result, little traction has been gained thus far for their wider implementation.

Traditionally, CEA evaluates the value of an intervention from a clinical and cost perspective, determining value as a trade-off between cost and health benefit (Canadian Institute for Health Information (CIHI), 2022; Guidelines for the economic, 2017). The structured nature of CEA contributes to its appeal, as it offers a quantitative and reproducible method of analysis standardized across different disease areas and technologies for decision-makers, who are concerned largely with extracting the maximum value for treatments provided for any given condition. Herein, however, lies one of the major drawbacks of the traditional approach to CEA: its restrictive nature fails to capture the additional elements of values that do not fall precisely within these standard, well-defined parameters (Willke et al., 2018; Garrison et al., 2019; Neumann et al., 2022). The quality-adjusted life year (QALY), which is considered by many in the HTA field as the cornerstone of traditional CEA and one of the two drivers of CEA results (along with survival), is seen by others as inherently flawed and based largely on sometimes unfounded assumptions; as such, this compromises its ability to provide a meaningful calculation of an intervention’s value (Nord et al., 2009; Torbica et al., 2018; Caro et al., 2019; Rand and Kesselheim, 2021).

With this background in mind, this research aimed to identify if the additional elements of value previously described for consideration in HTAs have been proposed in modified economic modelling techniques or other deliberative approaches. In theory, various methodologies have been suggested to remedy the drawbacks of the current CEA approach. These methodologies range from slight alterations to QALY modifiers that consider additional elements of value without dramatically altering the current structure to completely new methodologies that attempt to maintain the objectivity of the CEA approach while incorporating expanded concepts of value (Asaria et al., 2016; Garrison et al., 2019). A commentary by Caro et al., 2019 (Caro et al., 2019) provided a critical summary of alternative approaches to QALYs that expand the measure of benefit/value of new technologies and help further deliberations on determining aspects of technology’s value. To supplement the arguments noted in this commentary, and to continue the discussion on how the new HTA era should focus on creating an equitable, efficient, and high-quality health system (O'Rourke et al., 2020a), this review aimed to identify and describe the expanded economic analyses beyond the traditional CEA approach by incorporating additional elements of a technology’s value in modelling approaches.

## Materials and methods

A pragmatic literature review using reproducible criteria was conducted to capture relevant peer-reviewed articles. Reporting was guided by the Preferred Reporting Items for Systematic Review and Meta-Analyses statement (Page et al., 2021). The research question followed the Sample, Phenomenon of Interest, Design, Evaluation, Research (SPIDER) format (Library UoC, 2022): how have assessments of value (beyond clinical and cost estimates) for health technologies been incorporated in recent modelling approaches and deliberative processes? A structured database search for publicly available literature published in English from 2012 to the present was conducted in Embase and MEDLINE on 24 March 2023 (see Appendix for complete search strategy). As the HTA process is rapidly evolving across many countries and “value” may be defined differently across cultures and healthcare systems, the review was not restricted by geography. Prior to commencing screening, a calibration exercise among reviewers was conducted on a random sample of 50 articles. Screening of titles/abstracts was carried out in the DistillerSR platform (Evidence Partners Incorporated; Ottawa, Canada) by a single reviewer with a second reviewer screening 15% of excluded articles as a quality check. The same approach was used for full-text screening. Eligible studies were required to meet all the following criteria: published following a peer-review process; discussed current HTA value frameworks in the context of CEA; provided new or expanded definitions of value; and discussed new modelling approach (es) to HTA. Studies that focused on disease-specific, value-based frameworks, solely on patient experiences, or strictly on economic modelling with no reference on how additional value elements can be incorporated were excluded. No grey literature sources or commentaries/editorials were considered for inclusion. Conference abstracts were excluded given the limited information provided.

Data extraction of included studies was carried out in a pre-specified template by a single reviewer and validated by a second reviewer. Data were extracted on publication characteristics, key themes, limitations in existing CEA approaches, and new recommendations for incorporating value within CEA. Each eligible article was evaluated based on three broad criteria: did the article comment on the suitability of the current HTA methodology; did the article discuss what aspects could or should be added to the current approach; and did the article discuss new methods for evaluating therapies? Included studies were categorized based on their recommendations for HTA agencies. The three main areas of methodology were: modifications to the current CEA approach (modified CEA: mCEA), which can include variations such as multi-criteria decision analysis (MCDA); and alternate approaches, such as discrete choice experiments (DCE). Within these frameworks, several sub-methodologies exist, such as distributional CEA (DCEA), augmented CEA (ACEA) and extended CEA (ECEA) within the mCEA framework; and different variations of current MCDA methods (Table 1).

**TABLE 1:** Summary of current traditional and expanded modelling approaches.

Modelling approach		Description
CEA	Traditional CEA Zamora et al., 2021	Uses the incremental cost-effectiveness ratio, which measures the costs incurred by the health system per quality adjusted life year gain when a new treatment or medical technology is used
Modified CEA	Augmented CEA Zamora et al., 2021	An extension of CEA that includes novel elements of value (e.g., insurance value, option value, and the value of hope) and considers trade-offs among them. This approach attaches a monetary value to all health gains
	Distributional CEA Cookson et al., 2017; Diaby et al., 2021	Focuses on the distributions of health effects (health gains/disease burden) associated with healthcare interventions at both population (societal) and subgroup (e.g., sex, race/ethnicity) levels as well as the distribution of health opportunity costs per equity-relevant sociodemographic variables and per disease categories. Decision making considers the trade-offs between improving total population health and reducing unfair health inequality.
	Extended CEA Cookson et al., 2017	Assesses the distribution of both health benefits and financial risk protection benefits and considers financial benefits of policies considering out-of-pocket payments in certain geographies
MCDA	Traditional MCDA Baltussen et al., 2017	Involves a structured and rational decision-making approach informed by evidence on multiple criteria that uses quantitative scores to choose, rank, select options
Modified MCDA	Equitable MCDA Diaby et al., 2021	Explicitly considers multiple criteria including the impact of treatment on health equality
	Qualitative MCDA DiStefano and Levin, (2019)	Incorporates qualitative considerations into MCDA by considering decision makers’ opinions on the importance of each criterion while prioritizing interventions and/or subgroups, as opposed to solely relying on quantitative scores
	Reflective MCDA Goetghebeur and Cellier, (2018)	Focuses on compassionate care and assumes that decision makers reflect on the goals of the analysis and whether those goals align with a compassionate care approach while considering both quantitative and qualitative factors
	Advance value tree Angelis and Kanavos, (2017)	A modified MCDA that uses three criteria levels to measure value across five domains (burden of disease, therapeutic impact, safety profile, innovation level, and socioeconomic impact)
DCE	Traditional DCE Ngorsuraches, (2021)	Involves participants sequentially choose between hypothetical options to make decisions on choices of treatment or healthcare service based on attributes such as efficacy, side effects, and costs
Modified DCE	Latent class DCE Ngorsuraches, (2021)	Incorporates a latent class model to derive the value of equity
	Quantum choice DCE Ngorsuraches, (2021)	Incorporates equity attributes for individual alternatives in choice tasks to derive the value of equity

Abbreviations: CEA, cost-effectiveness analysis; DCE, discrete choice experiment; MCDA: multi-criteria decision analysis.

## Results

### Study eligibility

**FIGURE 1:**
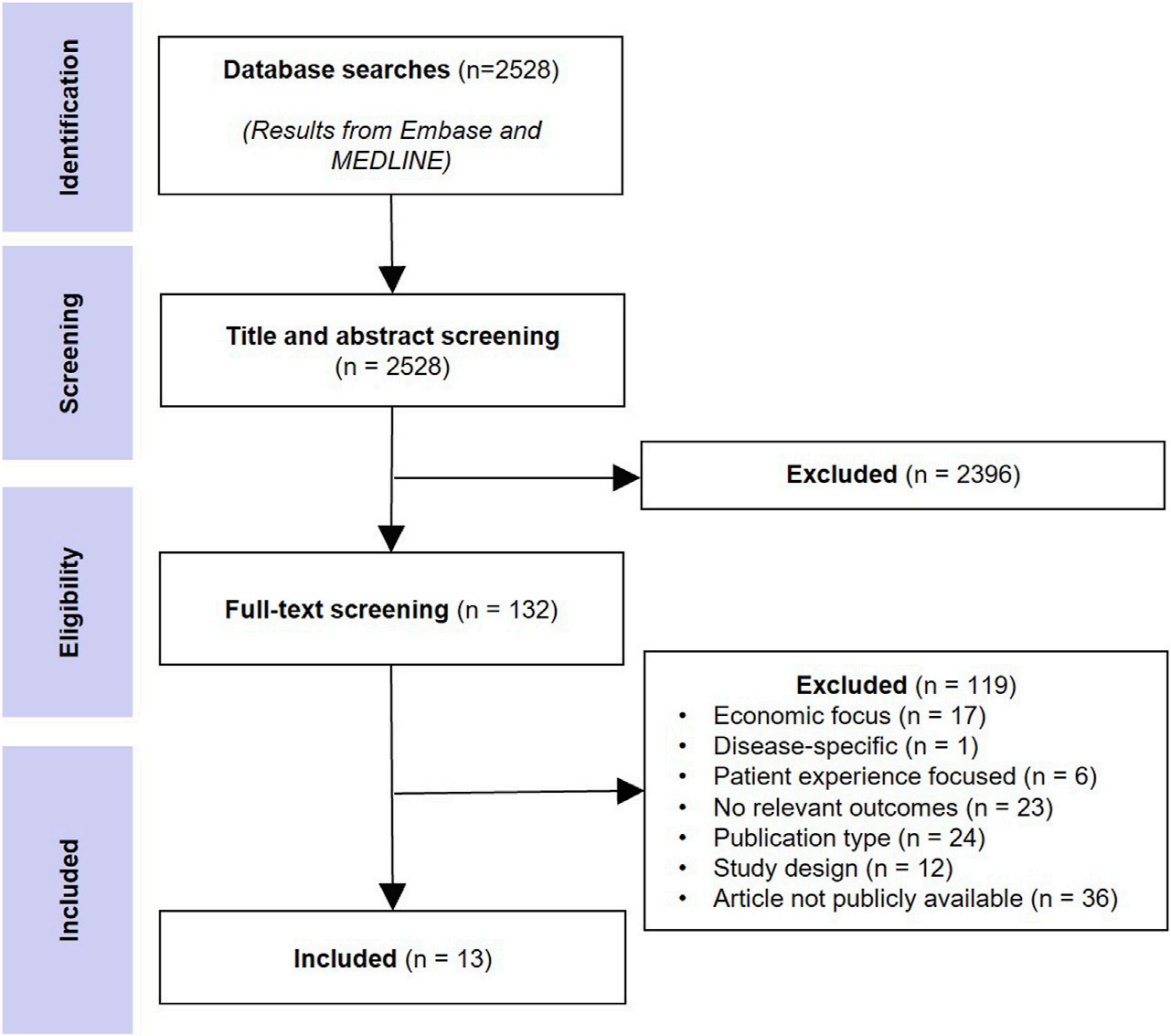
PRISMA diagram detailing literature search results and subsequent review process.

The database searches returned 3,614 records and after removing duplicates, 2,528 unique records were screened at the title/abstract level, and 132 were selected for full-text screening. Thirteen peer-reviewed studies that provided recommendations on new approaches to HTA in the context of CEA were included. Figure 1 shows the literature selection procedure.

### Description of included publications

**FIGURE 2:**
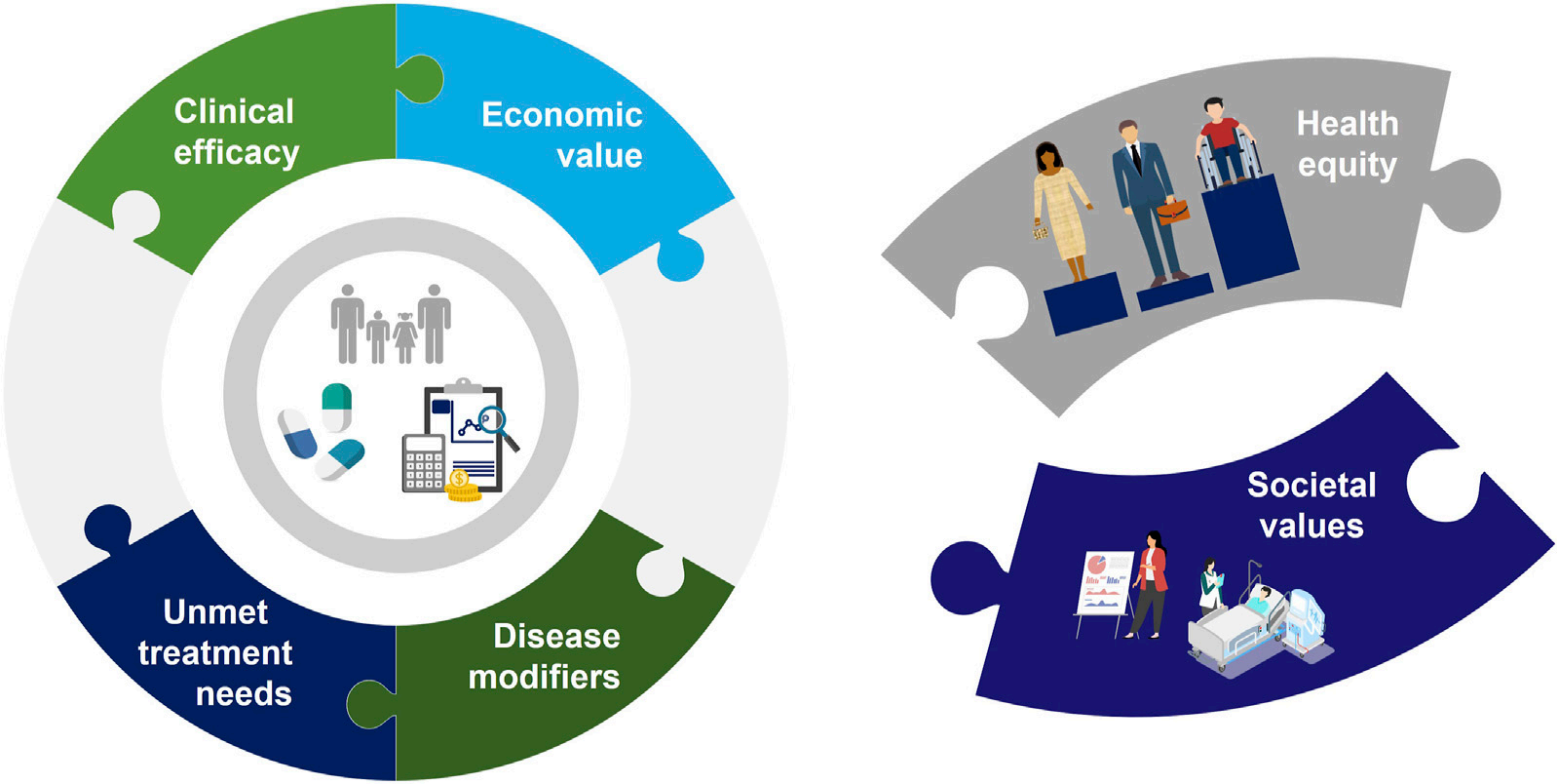
The “Value Puzzle” illustrates the existing aspects of CEA (clinical efficacy, economic value, disease modifiers and unmet treatment needs) but also highlights the missing aspects of the current system (health equity and societal values).

Ten of the 13 included articles were literature review articles offering expert opinion while three were reports from multistakeholder workshops or committees. Six articles specifically discussed at least one additional element to the current HTA (clinical and cost-effectiveness) value paradigm. Societal values and health equity were identified as the top two core pillars where the current CEA paradigm is wanting, with authors across these publications generally recommending an expansion of the definition of value within CEA to include these broad aspects (Figure 2).

Societal values were the most identified elements, mentioned in four articles (Dionne et al., 2015; Phelps and Madhavan, 2017; Pearson et al., 2019; Diaby et al., 2021). Societal aspects encompass a relatively broad spectrum of elements, but all authors agreed that the impact of disease on the patient is central to these societal considerations. For example, in the context of potentially curative treatments (Pearson et al., 2019), considerations of disease severity, its rarity, and the potential for a cure to extend life or limit the burden of illness (especially in children), as well as the value of hope and real option value offered by these treatments should be considered. The impact of productivity is also considered an important aspect to add to CEA (Dionne et al., 2015), as patients’ contributions to society are directly related to their health and wellness. Above all, the perspectives of all relevant parties, labelled as the “5Ps” (patients, providers, payers, producers, and planners) are encouraged to be considered by decision-makers (Dionne et al., 2015; Phelps and Madhavan, 2017).

Health equity was identified in four articles (Dionne et al., 2015; Cookson et al., 2017; Goetghebeur and Cellier, 2018; Diaby et al., 2021) as an important factor that is largely lacking in the existing HTA frameworks. Equity is defined by the World Health Organization as: “the absence of unfair, avoidable or remediable differences among groups of people, whether those groups are defined socially, economically, demographically, or geographically or by other dimensions of inequality (e.g., sex, gender, ethnicity, disability, or sexual orientation)” (Organization WH, 2022; Sarri, 2022). Inequity is thus evident in circumstances where a deficit in one of these areas affects access to affordable care, which is limited in one or more marginalized groups. Cookson et al., 2017 (Cookson et al., 2017) made equity the core of their argument for new approaches in HTA analyses, focusing on the trade-offs required to ensure health equity and the net equity impact of HTA decisions. They argued that the tools for health equity analysis do exist (i.e., who gains and who loses in policy decisions) and that assessing the equity trade-offs should be incorporated into existing CEA methods. Similarly, Goetghebeur et al., 2018 (Goetghebeur and Cellier, 2018) framed equity as central to an approach based on the application of compassionate care concepts, where ethical considerations are contemplated by decision-makers to maximize equity and sustainability. Diaby et al., 2021 (Diaby et al., 2021) and Dionne et al., 2015 (Dionne et al., 2015) discussed equity from the patient’s perspective, with patient demographics and a lack of patient heterogeneity in clinical studies mainly contributing to inequity in health assessment. The low representation of minority groups in clinical studies is suggested to under-represent the effect of therapies on these populations, thus contributing to decreased availability of treatments for these patients. Consideration of individual patient needs (i.e., patient preferences) and fairness in how health-economic decisions are made (i.e., balancing population and individual priorities while considering patient age, alternate treatments, and equity across different jurisdictions and populations) are additional dimensions of health equity domain (Dionne et al., 2015). In summary, researchers have long argued for societal values and equity considerations to be incorporated into existing HTA frameworks. In the context of societal values, it was argued that the impact of disease and its characteristics on patients and their productivity should also factor into decision-making. Similarly, patient preferences, addressing the needs of underrepresented groups, and ensuring access to affordable care are central to including equity considerations in HTA frameworks (Dionne et al., 2015; Diaby et al., 2021; Sarri, 2022).

### Summary of mCEA or new modelling approaches

**FIGURE 3:**
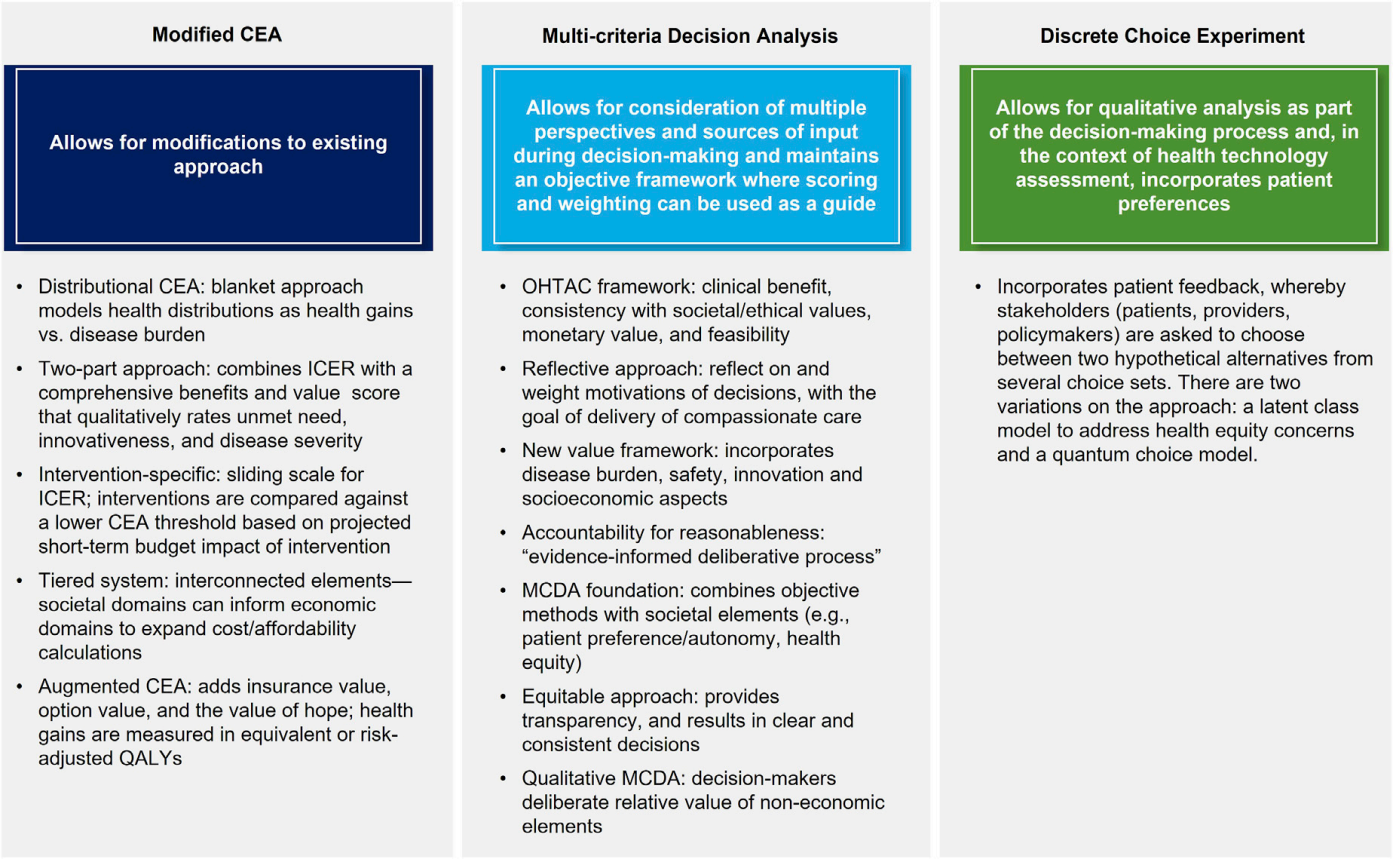
Overview of approaches recommended in the literature.

All eligible studies provided recommendations on new or modified approaches to HTA and CEA, which are mainly grouped as follows: mCEA; adoption of MCDA methods for CEA; and methods taking alternate approaches, such as DCE (Figure 3). The main theses and conclusions of the included peer-reviewed articles are summarized in Table 2.

**TABLE 2:** Summary of eligible articles.

Author, publication date	Modelling approaches	Targeted value elements	Themes noted
Diaby et al., 2021	MCDA, mCEA (distributional), CBV score	Health equity	The authors examine the current landscape regarding value frameworks in HTA, with a focus on the impact of current methodologies on health equity. They highlight the lack of diversity among most patient populations in RCTs and the fact that current frameworks largely ignore patient heterogeneity, complex demographic factors, and access to care. Three approaches are proffered to address these shortcomings: a 2-part HTA incorporating traditional HTA methods with a CBV score; a distributional CEA method; and equitable MCDA methods.
Ngorsuraches, (2021)	DCE (latent, quantum)	Health equity	Drawbacks of the current HTA methodologies are discussed and two new methods of assessment, based on DCEs are provided. One model utilizes a latent class model to address health equity in value assessment; the other uses a quantum choice model.
Zamora et al., 2021	mCEA (augmented), MCDA	Societal values (value of hope, value of insurance)	A comparison between a modified CEA approach (augmented CEA, ACEA) and an MCDA approach to HTA decisions is explored including an examination of the trade-offs between financial loss and healthcare gain. A context of insurer coverage for healthcare innovations, i.e., new medical technologies, is used.
DiStefano and Levin, (2019)	MCDA (qualitative)	Health equity	The authors discuss current drug prescribing guidelines and how the addition of CEA concepts in the guidelines may help promote improved equity in health. They discuss several concepts within traditional CEA methods, including MCDA approaches, arguing that a *qualitative* MCDA approach may be preferred. A qualitative approach forgoes the aggregation of scores and allows decisions to include deliberations amongst decision-makers.
Pearson et al., 2019	mCEA	Societal values (real option value, value of hope, value of insurance, value of potential cure)	The authors outline drawbacks of current “utilitarian” HTA frameworks, including the inability of current methods to account for social values such as disease severity and rarity, burden of illness and the ability of curative treatments to extend life, especially that of children. They suggest modifications to the current CEA approach, such as adopting a “sliding scale” for the ICER and capping drug prices based on willingness-to-pay metrics.
Krahn et al., 2018	MCDA	Social values (quality, evidence, effectiveness, equity, population health, collaboration)	A summary of the OHTAC framework is presented. An audit of the existing HTA methodologies is presented and recommendations for future assessments are made, including a focus on 4 key attributes: overall clinical benefit; consistency with expected societal and ethical values; value for money; and feasibility of adoption into the healthcare system.
Goetghebeur and Cellier, (2018)	MCDA (reflective)	Compassionate care	A new approach to CEA based on MCDA methods is discussed. Central to the approach is the concept of compassionate care, the underlying impetus of healthcare. The method involves analysis of quantitative (e.g., disease severity) and qualitative (e.g., health system capacity) factors but also allows for an opportunity for reflection on the goals of the analysis and whether those goals align with a compassionate care approach.
Angelis and Kanavos, (2017)	MCDA	Disease burden, therapeutic value, safety, innovation, socioeconomic value	The authors discuss the current CEA approaches, identify drawbacks and present a new approach that includes evaluation of the burden of disease, the level of innovation of the intervention, its ease of use and its socioeconomic value. A decision-tree model is utilized to provide guidance in decision-making.
Kristensen et al., 2017	mCEA	Organizational aspects, ethical aspects, patient/social aspects	This study presents a new approach for CEA based on a 10-year effort by the European HTA agencies. It discusses the application of a model designed for both full and rapid assessments. Nine domains are outlined, all of which are part of the full assessment but only four of which are part of the rapid assessment.
Baltussen et al., 2017	MCDA	Ethical issues	The authors summarize the current landscape of CEA and presents a new approach, utilizing MCDA methods. The new method importantly includes stakeholder deliberation to facilitate learning and combines MCDA methods with accountability for reasonableness techniques into a new approach dubbed an “evidence-informed deliberative process.” The responsibilities of the various HTA parties in implementing the new approach are discussed in detail.
Phelps and Madhavan, (2017)	MCDA	Patient-centered variables	A summary of the shortcomings of the current CEA methods is presented, highlighting several areas where current methods are lacking. A new approach is recommended, based on MCDA methods, which provides scores for aspects of decisions for oncology patients such as the likelihood of hair loss or nausea with certain treatments. The authors focus on the concept of perspective and highlight the importance of perspective from multiple perspectives, including that of the patient, the provider, the payer, the producer (manufacturer) and the planner (the “5Ps”).
Cookson et al., 2017	mCEA (distributional, extended)	Health equity	The authors focus on the costs and benefits of CEA in the context of health equity and equitable access to treatment. A new approach to CEA using extended CEA or distributional CEA as the preferred methods of analysis is recommended. As part of this approach, the roles of equity impact analysis and equity trade-off analysis in decision-making are explored.
Dionne et al., 2015	MCDA	Societal benefit, health equity, patient autonomy, innovation	The authors identify several areas where the current CEA methodology is lacking and discuss additional aspects that should be included in future, including factors that address societal values. Several factors are deemed important when considering new aspects, including comparative effectiveness, adoption feasibility, patient autonomy, societal benefit, equity, innovation and disease prevention. A new methodology using MCDA techniques is recommended and discussed in the context of rare diseases and end-of-life decisions.

Abbreviations: ACEA, augmented cost-effectiveness analysis; CBV, comprehensive benefits and value; CEA, cost-effectiveness analysis; DCE, discrete choice experiment; HTA, health technology assessment; ICER, incremental cost-effectiveness ratio; MCDA, multi-criteria decision analysis; mCEA, modified cost-effectiveness analysis; OHTAC, ontario health technology advisory committee; RCT, randomized controlled trial.

### mCEA

Four articles (Kristensen et al., 2017; Pearson et al., 2019; Diaby et al., 2021; Zamora et al., 2021) recommended mCEA as an expanded CEA method to incorporate additional value elements, albeit their suggestions differed considerably. Kristensen et al., 2017 (Kristensen et al., 2017) summarized the results of a decade-long analysis of HTA methods by the European Network for HTA (EUnetHTA), which recommends a tiered system that accounts for typical domains such as effectiveness, safety, and health economics but also includes domains addressing social, patient, legal, and organizational elements. EUnetHTA identified nine core elements that should be considered by HTA agencies and, as part of its tiered approach, delineated between a rapid relative effectiveness assessment (REA) for interventions requiring a short turnaround and a full, comprehensive assessment for all other interventions. The REA would focus on basic clinical elements (e.g., health problem identification, intervention description, safety, and clinical effectiveness), while the full assessment would add elements such as costs, ethical analysis, organization impact, patient/societal aspects, and legal aspects. The core elements are designed to be interconnected, such that the costs/economics domain can draw information from other domains (e.g., organizational or patients/social aspects) to expand the calculation of cost and affordability. This allows for a more comprehensive and nuanced analysis that better incorporates non-traditional elements.

Diaby et al., 2021 (Diaby et al., 2021) offered two recommendations on mCEA methods: the DCEA, and a two-part appraisal that augments incremental cost-effectiveness ratio (ICER) with a comprehensive benefits and value (CBV) score. DCEA refers to a blanket approach to technology assessment that models health distributions as a comparison of health gains vs. disease burden. This approach allows for analysis of health interventions at the population (i.e., societal) and demographic subgroup (e.g., sex, race) levels, enabling an analysis of health gains in the context of sociodemographic variables, which inevitably incorporates elements of equity as defined by these variables. Health gains and losses can thus be analyzed based on individual sociodemographic variables and/or by disease category. Under this proposed approach, decision-makers are asked to make trade-offs between decisions that would improve the overall health of the population and those that would reduce inequity in healthcare availability among specific patient subgroups. The two-part approach combines ICER threshold with a CBV score, allowing for a more robust analysis that considers quantitative and qualitative assessment factors. The CBV score is a composite, qualitative rating calculated using elements such as innovativeness, disease severity, and unmet need (Goldman et al., 2010; Diaby et al., 2021) which provides a more holistic assessment of the non-economic aspects of a given intervention.

Zamora et al., 2021 (Zamora et al., 2021) examined the potential of ACEA to incorporate additional individual value elements such as insurance value, option value, and the value of hope to the traditional ICER approach. Any health gains from new elements are measured in equivalent or risk-adjusted QALYs. A hierarchical approach is then used to calculate the final aggregate impact of an intervention, beginning with the incremental QALY and then incorporating QALY equivalents for new elements. Ultimately, final decisions on technologies’ value are made through consideration of the trade-offs among the elements, as gains in one area may be associated with losses in another. The ability to quantify benefits/losses of an intervention in a common unit of measure (QALY) creates a single denominator in the calculation, which maintains an objective framework while incorporating elements that may traditionally be considered subjective. Additionally, the authors compared the ACEA and MCDA approaches and found them to be fairly similar, such that the choice between the two was largely pragmatic and thus their research question was left unresolved.

Finally, Pearson et al., 2019 (Pearson et al., 2019) discussed a more intervention-specific method that does not explicitly incorporate non-economic factors. They suggested several modifications, such as a sliding scale for the ICER, specifically for curative treatments, where interventions can be compared against a lower CEA threshold based on the projected short-term budget impact of the intervention. They further recommended adaptations including disallowing full credit for cost offsets for any interventions no longer required after a condition is cured, if that intervention itself was not cost effective; capping costs based on patient willingness to pay; and using shared savings, such that cost savings realized by curative treatments are shared between the innovator and the healthcare system. Fundamentally, their approach seeks to modify the calculations made during HTA but maintain the ability to objectively calculate costs and cost-savings, an approach not unlike that of MCDA in its desire to maintain a level of objectivity in decision-making.

### MCDA

Nine articles (Dionne et al., 2015; Angelis and Kanavos, 2017; Baltussen et al., 2017; Phelps and Madhavan, 2017; Goetghebeur and Cellier, 2018; Krahn et al., 2018; DiStefano and Levin, 2019; Diaby et al., 2021; Zamora et al., 2021) recommended the adoption of some version of MCDA as a method for future CEA. MCDA methods allow for consideration of multiple perspectives and sources of input during decision-making with the aim to maintain an objective framework where scoring and weighting can be used to guide the process (Koksalan et al., 2011). Krahn et al., 2022 (Krahn et al., 2018), summarized the findings of the Ontario Health Technology Advisory Committee which recommend a framework that includes four key attributes: consideration of overall clinical benefit, consistency with societal/ethical values, value for money, and feasibility. It is within this framework that they advocate for the use of MCDA methods, citing the Evidence and Values Impact on DEcision Making framework (EVIDEM) (Goetghebeur et al., 2008) as an approach that is being increasingly explored and should be considered in future HTAs. Goetghebeur (Goetghebeur and Cellier, 2018) extended this work to suggest the use of a reflective MCDA approach, where decision-makers can reflect on and weight the motivations of their decisions, bearing in mind that decisions should be made in a patient-centric manner, with an eye toward the delivery of compassionate care. Angelis et al., 2017 (Angelis and Kanavos, 2017) outlined a new value framework using MCDA methods as a foundation, which incorporates several key aspects such as burden of disease, therapeutic considerations, safety, innovation, and socioeconomic considerations. Their resulting decision-tree approach considers each of these elements, with subsequent downstream decisions made based on each one; the final decision is based ultimately on the cumulative impact of each element and decision. Baltussen et al., 2017 (Baltussen et al., 2017) combined MCDA methods with accountability for reasonableness concepts as part of a new approach that they refer to as an “evidence-informed deliberative process.” They categorized the traditional elements assessed by HTA agencies (e.g., safety, effectiveness, budget impact) as “general criteria” and advocated for the additional consideration of “contextual criteria,” which encompass more patient-centric or societal considerations. They recommended consultation with the public on what contextual elements may be important during an HTA. As such, a combination of quantifiable criteria and non-quantifiable (i.e., qualitative) criteria should be considered, with both ultimately being used as inputs into the deliberative process. Phelps, et al., 2017 (Phelps and Madhavan, 2017), Dionne et al., 2015 (Dionne et al., 2015), and Zamora et al., 2021 (Zamora et al., 2021) also advocated for the use of MCDA methods as a way to maintain objectivity in decision-making, while still taking into account societal elements such as patient preference/autonomy and health equity. Diaby et al., 2021 (Diaby et al., 2021) similarly suggested using MCDA methods as part of an “equitable MCDA” approach, one that is transparent and results in clear and consistent decisions. They suggested the importance of both the consideration of multiple criteria as well as the impact of a given treatment on health equality. Finally, DiStefano et al., 2019 (DiStefano and Levin, 2019) stressed the importance of qualitative MCDA (Baltussen et al., 2019) which, by forgoing aggregation of scores, allows decisions to include deliberation among decision-makers regarding the relative value of non-economic elements, thus maintaining transparency and allowing for more subjective incorporation of elements such as equity, rather than integration of those elements into more traditional or mCEA. This aims to maintain the objective nature of MCDA while allowing for subjective consideration in the decision-making process.

### DCE

One included publication specifically looked at the use of the DCE model for future HTA decisions (Ngorsuraches, 2021). The authors suggested that DCE allows for a qualitative analysis as part of the decision-making process and, in the context of HTA, incorporates patient preferences while all stakeholders involved (patients, providers, policymakers) are asked to choose between two hypothetical alternatives from a number of choice sets. As such, the prevalence and importance of equity to each stakeholder is determined in the initial stage, after which those equity elements can be incorporated into a choice model which, when applied, can be used to establish the value of equity. Two variations on the DCE approach include one which utilizes a latent class model to address concerns about health equity in value assessment, and another which utilizes a quantum choice model. The author did not express a preference for one method over another but noted that the use of either methods would address the inadequacies of current methodologies and help address health disparities and underrepresented patient populations.

## Discussion

In most countries, HTA remains anchored by CEAs, the cornerstone analysis when considering reimbursement of new therapies. The objective nature of the traditional CEA is seen as a benefit that lends itself to impartial decision-making although this method did not entirely prevent discrepancies in decision-making; however, there is increasing sentiment that the objective approach in fact marginalizes the subjective aspects of the healthcare assessment. The definition of “value” is a prime example of the drawbacks of the current system, as there is a growing opinion that value in HTA should be viewed through more than simply an economic lens. Health gains are not straightforwardly assessed, and several approaches have been proposed to define additional elements of value beyond the clinical and cost gains. Lately, there is an increased discontent with the inability of ICERs and QALYs to sufficiently capture the benefits valued by patients and societies overall when a new health technology is introduced (Caro et al., 2019). Although mainly the discussion so far has focused on defining these additional value elements, little effort has been put on demonstrating how these additional considerations can be implemented in modelling approaches to be used in the HTA context. To address this gap, the current study examined recent modelling approaches that included expanded or new definitions of value; two main analytical approaches were identified and advocated by most authors: a modification of the current CEA and the use of advanced decision-making techniques such as MCDA, both of which have merit. To date, however, no preferred method has been established for HTA adoption although several concerns have been raised regarding the implementation of MCDA as part of HTA decision-making (Marsh et al., 2018).

Despite these efforts, consensus on the most efficient and appropriate way to incorporate expanded definitions of value (and added benefit value frameworks) into current HTA in general and CEA methods in particular has remained elusive. One consensus finding from this review was that the current approach to CEA is lacking and that there are several elements–especially relating to the current narrow definition of value in CEA–that should be added to CEA methods going forward. These aspects represent missing pieces of the “Value Puzzle” (Figure 2) and illustrate the challenges assessors face in integrating these new factors into their decision-making. These factors have been identified by several groups, including the ISPOR Task Force, which summarized these concepts in the “Value Flower” (Lakdawalla et al., 2018; Willke et al., 2018; Garrison et al., 2019; Neumann et al., 2022). Generally, these missing aspects center around an expanded definition of value, one which includes more qualitative elements such as the ability of a treatment to provide hope to the patient (the value of hope), the value associated with extending life and opening possibilities for future treatments (real option value), the value of scientific discoveries and their wide applicability (scientific spillover), and more. These new elements reflect the prevailing opinion that the current approach, with its focus on cost-effectiveness and the use of metrics such as the ICER, is inadequate. However, these additional value elements may not transport at the same degree across disease areas and populations (Shafrin et al., 2021). Indeed, many are of the opinion that the central metric in these calculations–the QALY–is an inherently flawed metric built upon assumptions (Hall, 2020) which marginalizes the sickest in a population by presenting only an aggregate calculation of health (Caro et al., 2019). Indeed, many jurisdictions have begun to move away from the QALY, which has been outright rejected in Germany and Spain (Institute for Quality and Efficiency in Healthcare IQWiG, 2022) and remains largely unused in the United States (Neumann and Weinstein, 2010), France (Rumeau-Pichon and Harousseau, 2014), and some Latin American countries (Brixner et al., 2017). It is thus important to recognize the limitations of the QALY as a final, lone decision metric and that its use represents a first albeit limited step in the process of assessing value in pharmaceutical innovation. Clearly, the lack of enthusiasm for traditional CEA methods among HTA bodies and the feedback from patients necessitates a new approach to decision-making.

The current study identified two main themes recommended to address the shortcomings of the current system: adoption of either MCDA methods or modification of the current CEA approach. The former was advocated for in the majority of articles included in the review and has been widely discussed in the CEA space as a viable alternative for some time; however, it has failed to gain traction, at least in part due to its overly mechanistic nature (Kennedy, 2009; Baltussen et al., 2019) and tendency to ignore opportunity costs (Campillo-Artero et al., 2018; Marsh et al., 2018; Baltussen et al., 2019). Quite the reverse, the use of mCEA methods has been suggested as a viable avenue for change that simultaneously addresses concerns raised by Caro et al., 2019 (Caro et al., 2019), who suggested the current CEA methods continue to be utilized by HTA bodies mainly out of convenience, and due to the lack of a viable, proven alternative. Thus, one of the draws to modifying current methods is the fact that it does not stray far from the *status quo*. With infrastructure in place and decades of published decisions, a major change in methodology may not be palatable for key stakeholders. Some of the recommendations in the current review slightly revised the current approach but did not recommend major changes (Kristensen et al., 2017; Pearson et al., 2019; Diaby et al., 2021). As such, these recommendations perhaps do not do enough to address current concerns. Garrison et al., 2017 (Garrison et al., 2017) proposed the use of ECEA methods to incorporate the value of “knowing” into CEA, which broadly incorporates several elements identified as valuable in our review, including reducing uncertainty and incorporating insurance value, real option value and scientific spillover into CEA. Another approach that holds promise is the ACEA approach (Zamora et al., 2021) which, like ECEA, combines the known methodology while still incorporating robust definitions of value including the value of hope, real option value, and insurance value. Indeed, the summary of the ISPOR Special Task Force report (Garrison et al., 2020) advocated for the use of ACEA methods as a way to combine the known (and widely accepted) clinico-economic aspects of traditional CEA with a comprehensive list of qualitative elements reflecting the various definitions of value. That recommended method would allow for the consideration of additional value elements (insurance, disease severity, hope, and real option value) while also allowing concepts such as equity and the benefit of scientific spillover from new technologies to be incorporated into deliberations. These additional value elements can be part of the technologies scoping exercises and tailored to the patients’ preferences. While more research is needed to refine the methods, this approach shows promise and may best address the documented shortcomings of the current approach.

Beyond methodology, HTA agencies face many other challenges in their efforts to fairly evaluate new therapies. As environmental awareness and concern grows worldwide, HTA agencies will be required to include an evaluation of the impact of a health technology’s production, use, and disposal. Toolan et al. (Toolan et al., 2023) have recently summarized the challenges associated with this effort and identified several approaches that HTA agencies may adopt during their assessment, including republishing of data in the public domain, considering environmental data in parallel with health economic data, integrating environmental data into existing methodologies, or specific evaluation of technologies that may have minimal health benefits but claim environmental benefits with their use. From a more patient-centric perspective, patients’ perspectives and preferences have been suggested as important factors that warrant more attention in the HTA process. Several authors have referred to “The 5Ps” as important contextual considerations in HTA, namely, that the perspectives of many stakeholders–patients, practitioners, payers, producers and policymakers–must be part of any CEA (Phelps and Madhavan, 2017; Slejko et al., 2019; Hall, 2020; van Overbeeke et al., 2021). Incorporating patient preference and experience, and their perception of the quality of life amidst their illness, offsets the objective nature of the traditional CEA methods and theoretically allows for a more comprehensive assessment (Sarri et al., 2021). For example, factors important to the patient regarding the impact their diagnosis will have on those around them (Vrinzen et al., 2022) or life satisfaction should be considered in any assessment (Hall, 2020). Furthermore, a patient’s preference can be reflected in their willingness to pay for or undergo treatment based on whether that treatment can offer them hope for recovery (Peasgood et al., 2022). Several authors have noted that patients are more willing to pay for a “hopeful therapy”, with patients with cancer identified as those who prefer a therapy that has the possibility of a large therapeutic gain, even when the average response to that therapy may be similar to other options (Lakdawalla et al., 2012). As Hall comments: a patient who adapts to illness may live longer but may be less able to fight off future illness. Do the patient’s values change as they adapt to their disease? And how does the QALY account for this adaptation (Hall, 2020)? Administratively, the financial burden placed on healthcare systems will only continue to increase. Healthcare systems stretched thin by the recent COVID-19 pandemic face ongoing challenges in integrating costs for new therapies into an already strained system (Epstein et al., 2020; Information CIfH, 2022; Youn et al., 2022). However, recent trends such as the growing use of real-world evidence (RWE) in healthcare research in general and with it a concomitant uptake in the use of RWE in regulatory and HTA agency filings may provide the opportunity to unravel existing health inequalities that directly fit in the decision-making (Sarri, 2022). However, the potential of RWE to capture the direction and magnitude of impact a new health technology may have on health inequalities has not been fully explored (Goetghebeur and Cellier, 2018). Proposed checklists to guide HTA decision-makers include equity considerations in their assessment may help on this front (Benkhalti et al., 2021). The struggle for HTA staff to keep pace with evolving RWE methodological complexities adds to the challenges facing these agencies. This is especially true in cases of rare disease or where ethical concerns prevent the designing of placebo-controlled, two-armed studies (Thorlund et al., 2020; Ramagopalan et al., 2021; Popat et al., 2022). All told, the challenges facing HTA bodies are layered and complex. More case studies are needed to demonstrate how reliably these holistic value aspects can be integrated into HTA, although buy-in among assessors and researchers is also required, to facilitate the widespread use of new and expanded methodologies and the learnings from demonstration of case studies.

This study should be considered with the following limitations. The pragmatic nature of the search, while comprehensive, could have missed some relevant articles, although the broad nature of the search may mitigate this concern. Related is the decision to include only peer-reviewed articles in data/theme collection. Commentaries and/or editorials were excluded from this review, which may result in some valid recommendations regarding these modelling techniques being missed. However, any commentaries that were captured in the search were reviewed for relevant opinions and referenced in the discussion as appropriate. Finally, articles that focused on a specific disease were excluded, as the aim was to provide a broad overview of these modelling techniques. This may also result in missing some articles that may have offered valuable perspectives on this topic; however, the wider focus of the review may make the findings more broadly applicable and initiate some methodological discussion.

## Conclusion

This research demonstrated that modelling methods are being expanded from the traditional CEA approach to incorporate value elements with a more holistic view of what matters most to patients and society. Although the methods differ, a consensus exists on the need for HTA agencies to redefine “value” with a wider lens that looks at more than just the clinical and economic benefits of a new technology. Societal factors and health equity scored highly as additional value elements. Future efforts are needed to increase the confidence of stakeholders in the importance of “testing” these expanded CEAs approaches in case studies.
